# Non-invasive assessment of liver fibrosis by serum metabolites in non-human primates and human patients

**DOI:** 10.1016/j.isci.2023.107538

**Published:** 2023-08-03

**Authors:** Tianhang Feng, Chunyou Lai, Qiuyun Yuan, Wanchun Yang, Yutong Yao, Mengze Du, Deyuan Zhong, Sijia Wang, Qinyan Yang, Jin Shang, Ying Shi, Xiaolun Huang

**Affiliations:** 1 Department of Hepatobiliary and Pancreatic Surgery, Sichuan Academy of Medical Sciences, Sichuan Provincial People’s Hospital, School of Medicine, University of Electronic Science and Technology of China, Chengdu, China; 2 State Key Laboratory of Oral Diseases, National Center of Stomatology, National Clinical Research Center for Oral Diseases, West China Hospital of Stomatology, Sichuan University, Chengdu, Sichuan, China; 3 Department of Neurosurgery, West China Hospital, Sichuan University, Chengdu, China; 4 Sichuan Cancer Hospital & Institute, Sichuan Cancer Center, School of Medicine, University of Electronic Science and Technology of China, Chengdu, China

**Keywords:** Diagnostic technique in health technology, Nonclinical topic

## Abstract

Liver fibrosis, a rising cause of chronic liver diseases, could eventually develop into cirrhosis and liver failure. Current diagnosis of liver fibrosis relies on pathological examination of hepatic tissues acquired from percutaneous biopsy, which may produce invasive injuries. Here, for non-invasive assessment of liver fibrosis, we applied comparative multi-omics in non-human primates (rhesus macaques) and subsequent serum biopsy in human patients. Global transcriptomics showed significant gene enrichment of metabolism process, in parallel with oxidative stress and immune responses in fibrotic primates. Targeted metabolomics were concordant with transcriptomic patterns, identifying elevated lipids and porphyrin metabolites during hepatic fibrosis. Importantly, liquid biopsy results validated that specific metabolites in the serum (e.g., biliverdin) were highly diagnostic to distinguish human patients from healthy controls. Findings describe the interconnected transcriptional and metabolic network in primate liver fibrosis and provide potential indices for non-invasive detection of liver fibrosis in humans.

## Highlights

• Establishment of a robust liver fibrosis model in rhesus macaques
• Comprehensive exploration of transcriptomic/metabolomic signatures of liver fibrosis
• Non-invasive serum biopsy providing a potential approach for evaluating liver fibrosis
• Preliminary evidence for biliverdin as a potential liver fibrosis biomarker

## Graphical abstract

**Figure:**
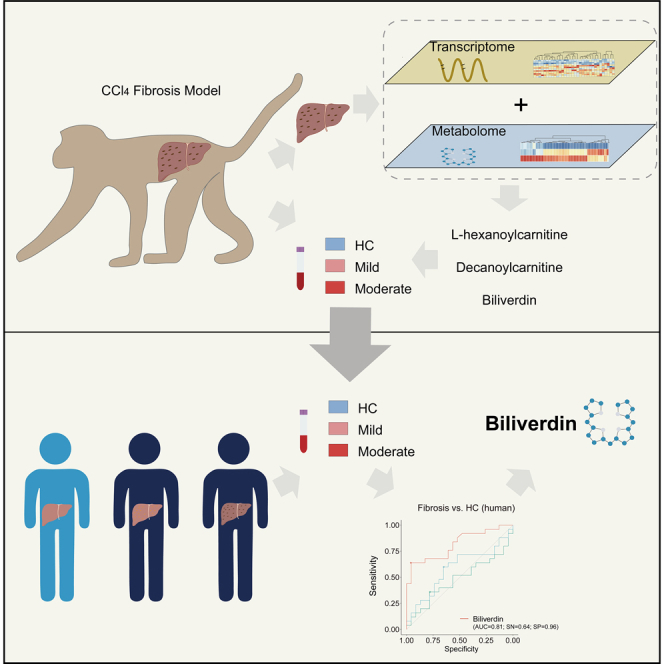

## Introduction

Liver fibrosis, featured by excessive accumulation of extracellular matrix (ECM), causes hepatic dysfunction and builds up a scar microenvironment in the liver.^1^ Pronounced fibrosis is one of the leading complications of multiple liver diseases, ranging from cirrhosis to liver failure.^2^ Accumulating evidence underscores that liver fibrosis is reversible after removal of factors that induces fibrogenesis.^3^ Therefore, liver fibrosis at early stages is suitable for clinical interventions once diagnosed. In practice, the gold standard method for diagnosis of suspected liver fibrosis requires histological assessment of liver samples obtained from percutaneous biopsy.^4^ However, liver biopsy is invasive and may produce an additional risk to patients by bleeding, infection, or accidental injury in the liver.^5^ In recent years, non-invasive methods were developed to assess liver diseases. Particularly, serum-based liquid biopsy is safe and convenient^6^ and thus preferable for the non-invasive detection of liver fibrosis.

Liver fibrosis is a dynamic process that accompanies with metabolic reprogramming.^7^ The notion of altered metabolism at the apex of initiation and progression in liver fibrosis highlighted a potential benefit of non-invasive diagnosis using metabolites in liver and serum samples.^8^ However, initial attempts to study the serum metabolites for liver fibrosis assessment were mostly based on rodent models, which may inevitably introduce marked species differences.^9^

In this study, we employed a comprehensive multi-omics analysis to elucidate the intricate interplay of transcriptomic and metabolomic profiles in our non-human primate (NHP) models of liver fibrosis. Moreover, we discerned distinct metabolic signatures from fibrotic primates, which were further evaluated to validate their diagnostic potential in human patients.

## Results

### Generation and validation of the liver fibrosis model in NHPs

**Figure 1:**
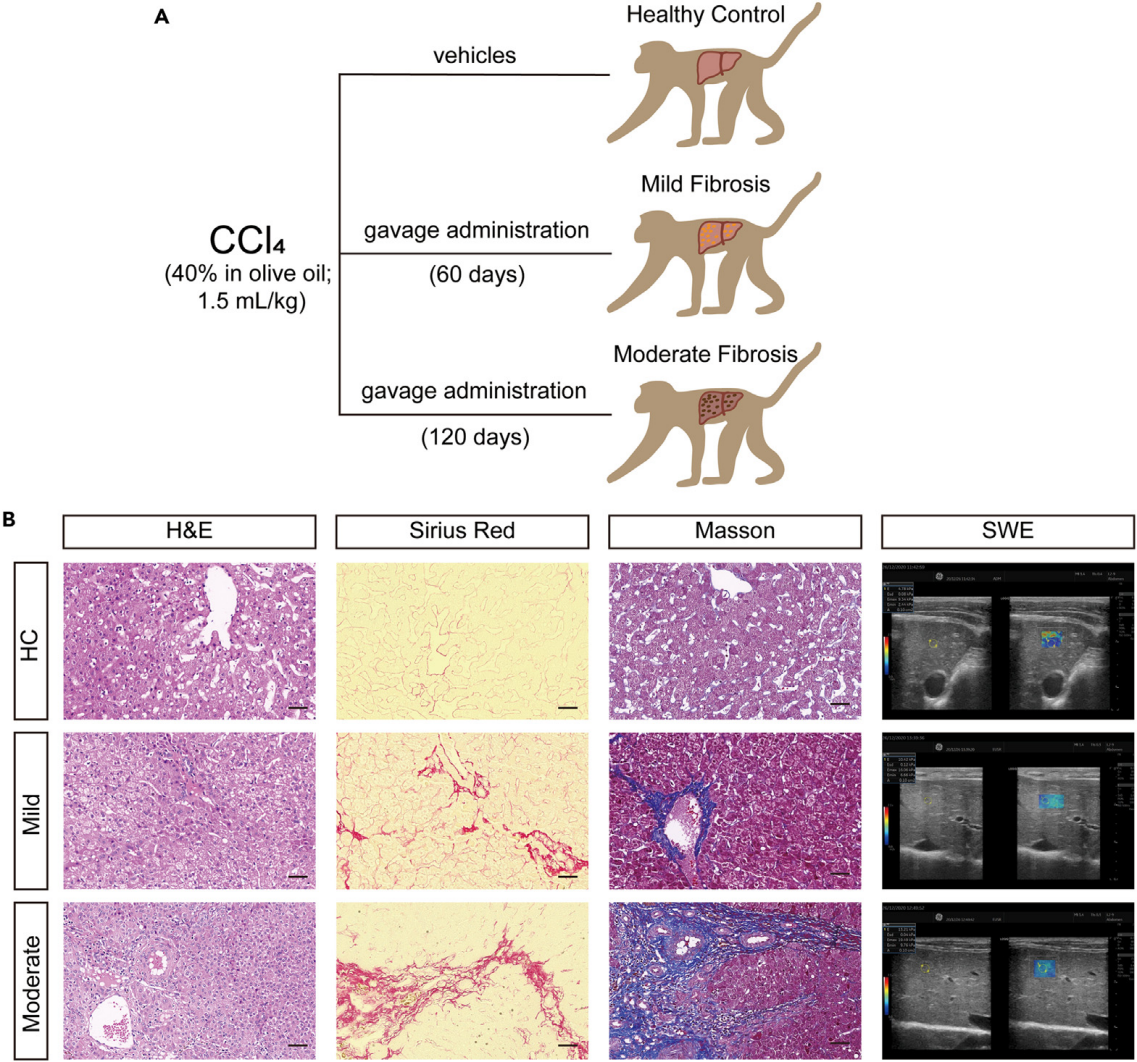
Characterization of liver fibrosis in non-human primates (A) Schema illustrating the strategy to construct the liver fibrosis model in rhesus macaques by CCl_4_ treatment through gavage administration (40% in olive oil). (B) Histological and shear-wave elasticity (SWE) images showing impaired hepatic structure and collagen deposition in the parenchyma of the liver of fibrotic primates treated with CCl_4_. Scale bar, 50 μm.

Given the substantial differences between primates and rodents, we generated an NHP model using rhesus macaques (*Macaca mulatta*) by carbon tetrachloride (CCl_4_) treatment that mirrors structural and functional damages of human liver fibrosis (Figure 1A and Table 1).^10^ The definition of mild and moderate fibrosis was based on the histochemical staining and shear-wave elastography (SWE) (Figure 1B). Images of hematoxylin and eosin (H&E) staining showed pronounced morphological alterations in the liver parenchyma, including disruption of tissue architecture, large fibrous septa formation, and extracellular fiber extension (Figure 1B, **left panel**). More detailed analysis of liver bridging fibrosis was confirmed by collagen deposition, as determined by Sirius red staining and concomitant increasing signals of Masson staining (Figure 1B, **middle panels**). Moreover, the Doppler color ultrasonography imaging quantified the stiffness of liver parenchyma, showing the increased values of SWE (Figure 1B, **right panel**). All these results confirmed the occurrence of liver fibrosis in rhesus macaques.

**Table 1:** Basic information of the rhesus macaques

	HC (n = 3)	Liver Fibrosis Monkeys (n = 9)		p value^a^
		Mild (n = 3)	Moderate (n = 6)	
Laennec scores	0	1	2	
SWE (kPa)	5.08 ± 0.605	6.76 ± 0.68	7.35 ± 0.81	0.006
Body weight (kg)	10.16 ± 0.91	7.83 ± 1.85	10.05 ± 2.14	1.000
Alanine transaminase (ALT, U/L)	16.83 ± 7.98	21.98 ± 9.28	76.27 ± 29.98	0.003
Aspartate aminotransferase (AST, U/L)	28.88 ± 13.00	34.167 ± 3.80	59.43 ± 6.31	0.007
Gamma-glutamyl transferase (GGT, U/L)	89.47 ± 21.11	80.33 ± 18.45	74.50 ± 11.54	0.511
Triglyceride (TG, mmol/L)	0.51 ± 0.14	0.28 ± 0.49	0.36 ± 0.07	0.087
Total cholesterol (TC, mmol/L)	3.39 ± 0.89	3.28 ± 0.95	3.06 ± 0.44	0.886
Total proteins (TP, g/L)	72.57 ± 8.28	64.30 ± 5.83	59.90 ± 5.98	0.067
Albumin (ALB, g/L)	46.10 ± 6.49	38.13 ± 7.54	41.55 ± 4.19	0.631
Urea (UREA, mmol/L)	8.26 ± 1.79	8.68 ± 1.02	7.29 ± 2.42	0.890
Cholinesterase (CHE, kU/L)	8.10 ± 1.88	10.89 ± 3.37	8.60 ± 3.17	0.994
Total bile acids (TBA, μmol/L)	4.73 ± 1.16	13.87 ± 15.58	2.77 ± 2.18	0.978
Creatinine (CREA, μmol/L)	83.97 ± 21.06	70.47 ± 24.95	75.11 ± 10.12	0.863
Uric acid (UA, μmol/L)	5.83 ± 3.03	5.80 ± 3.05	5.38 ± 0.95	0.988
White blood cell count (WBC, 10ˆ9/L)	6.33 ± 2.58	3.80 ± 0.62	4.83 ± 1.38	0.530
Lymphocytes (10ˆ9/L)	3.07 ± 2.04	1.30 ± 0.10	1.62 ± 0.67	0.250
Monocytes (10ˆ9/L)	0.77 ± 0.29	0.33 ± 0.06	0.38 ± 0.10	0.022
Granulocytes (10ˆ9/L)	2.50 ± 0.35	2.17 ± 0.55	2.83 ± 1.07	0.933
Lymphocytes %	45.4 ± 12.60	34.13 ± 4.69	33.93 ± 9.043	0.304
Monocytes %	12.47 ± 1.94	8.83 ± 0.86	8.52 ± 1.33	0.010
Granulocytes %	42.13 ± 11.54	57.03 ± 4.35	58.38 ± 9.16	0.089
Red blood cell (RBC, 10ˆ12/L)	5.23 ± 0.30	5.57 ± 0.53	5.25 ± 0.59	1.000
Hemoglobin (HGB, g/L)	127.67 ± 10.26	144.67 ± 9.02	139.83 ± 13.59	0.460
Hematocrit (HCT, %)	38.07 ± 3.50	42.33 ± 2.08	40.22 ± 3.90	0.790
Mean corpscular volume (MCV, fL)	72.87 ± 2.85	76.40 ± 3.67	76.77 ± 2.10	0.198
Mean corpuscular hemoglobin (MCH, pg)	24.37 ± 1.01	26.00 ± 1.14	26.60 ± 0.76	0.021
Mean corpuscular hemoglobin concentration (MCHC, g/L)	335.33 ± 7.57	341.33 ± 8.08	347.17 ± 4.36	0.069
Red cell distribution width (RDW, %)	13.00 ± 0.95	12.37 ± 0.90	11.75 ± 0.53	0.113
Platelet count (PLT, 10ˆ9/L)	288.00 ± 82.87	342.67 ± 23.12	310.00 ± 89.12	0.973
Mean platelets volume (MPV, fL)	8.77 ± 0.32	8.07 ± 0.32	8.92 ± 0.97	0.990
Platelet distribution width (PDW)	16.10 ± 0.27	15.87 ± 0.06	16.28 ± 0.34	0.771
Plateletcrit (PCT %)	0.25 ± 0.06	0.28 ± 0.03	0.27 ± 0.06	0.947

a Indicated the p value of HC vs. Moderate.

### The transcriptional and metabolic alterations in the liver of fibrotic primates

**Figure 2:**
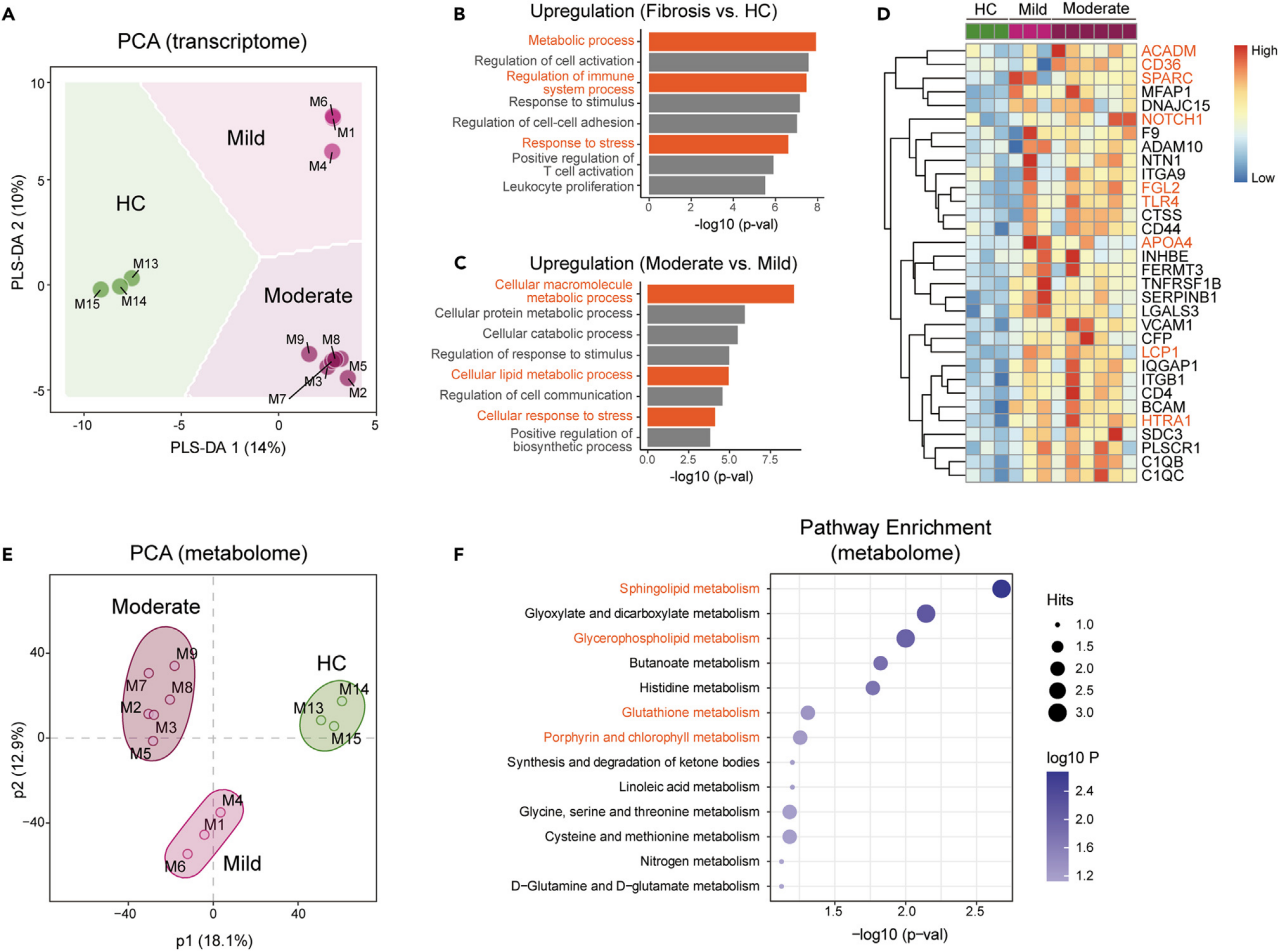
Transcriptomic and metabolomic signatures of liver fibrosis in non-human primates (A) Principal-component analyses (PCA) of the RNA-seq (transcriptome) data from fibrotic (mild and moderate) and HC monkeys. (B and C) Functional enrichment analyses using KEGG pathways for the gene clusters in fibrotic monkeys vs. HC (B) and moderate vs. mild fibrosis (C). Pathways are arranged by their -log_10_ (p value). (D) Heatmaps showing relative expressions of genes related to liver fibrosis. Data are represented as Z-score-normalized gene levels. (E) PCA of the metabolome data from fibrotic (mild and moderate) and HC monkeys. (F) Functional enrichment analyses using KEGG pathways for metabolite clusters in fibrotic monkeys vs. HC.

As a first step toward understanding the molecular signatures associated with liver fibrosis, we conducted unbiased transcriptome analysis in liver samples from fibrotic monkeys and healthy controls (HCs) by percutaneous biopsy. Principal-component analysis (PCA) revealed that the transcriptomic profiles of HC were separated from both Mild and Moderate groups (Figure 2A). By Kyoto Encyclopedia of Genes and Genomes (KEGG) pathway analysis, we found that cellular pathways, including metabolic process, responses to stress and immunes were robustly upregulated in Fibrotic groups compared to HC (Figures 2B and S1). Particularly, macromolecule pathways (e.g., lipids) showed consistent enrichment across Moderate vs. Mild fibrotic groups (Figure 2C), suggesting that lipid metabolism was tightly correlated with fibrotic progression. To further explore key molecular players in fibrotic monkeys, we identified the significant genes from lipid pathways (ACADM, NOTCH1, APOA4), ECM synthesis (SPARC, HTRA1, LCP1), and immune responses (CD36, FGL2, TLR4), which were progressively increased among HC, Mild, and Moderate groups (Figure 2D).

To verify the transcriptomic data and pinpoint critical metabolites that are involved in liver fibrogenesis, we set up to investigate the metabolic repertoire of fibrotic primates by liquid chromatography-mass spectrometry (LC-MS). PCA results showed that the metabolomic profiles of HC were consistently separated from either Mild or Moderate fibrosis groups (Figure 2E), in parallel with transcriptomic signatures. We next applied multivariate unbiased clustering analysis to identify the altered metabolites and mapped annotated pathways, showing that lipids (e.g., sphingolipids and glycerophospholipids), anti-oxidants (e.g., glutathione), and porphyrin metabolites belonged to the most aberrantly upregulated pathways in Fibrotic groups compared to HC primates (Figure 2F). Altogether, the transcriptomic and metabolomic profiles revealed that changes in hepatic metabolism are one of the significant events in fibrotic primates.

### The multi-omics analysis in the liver of fibrotic primates

**Figure 3:**
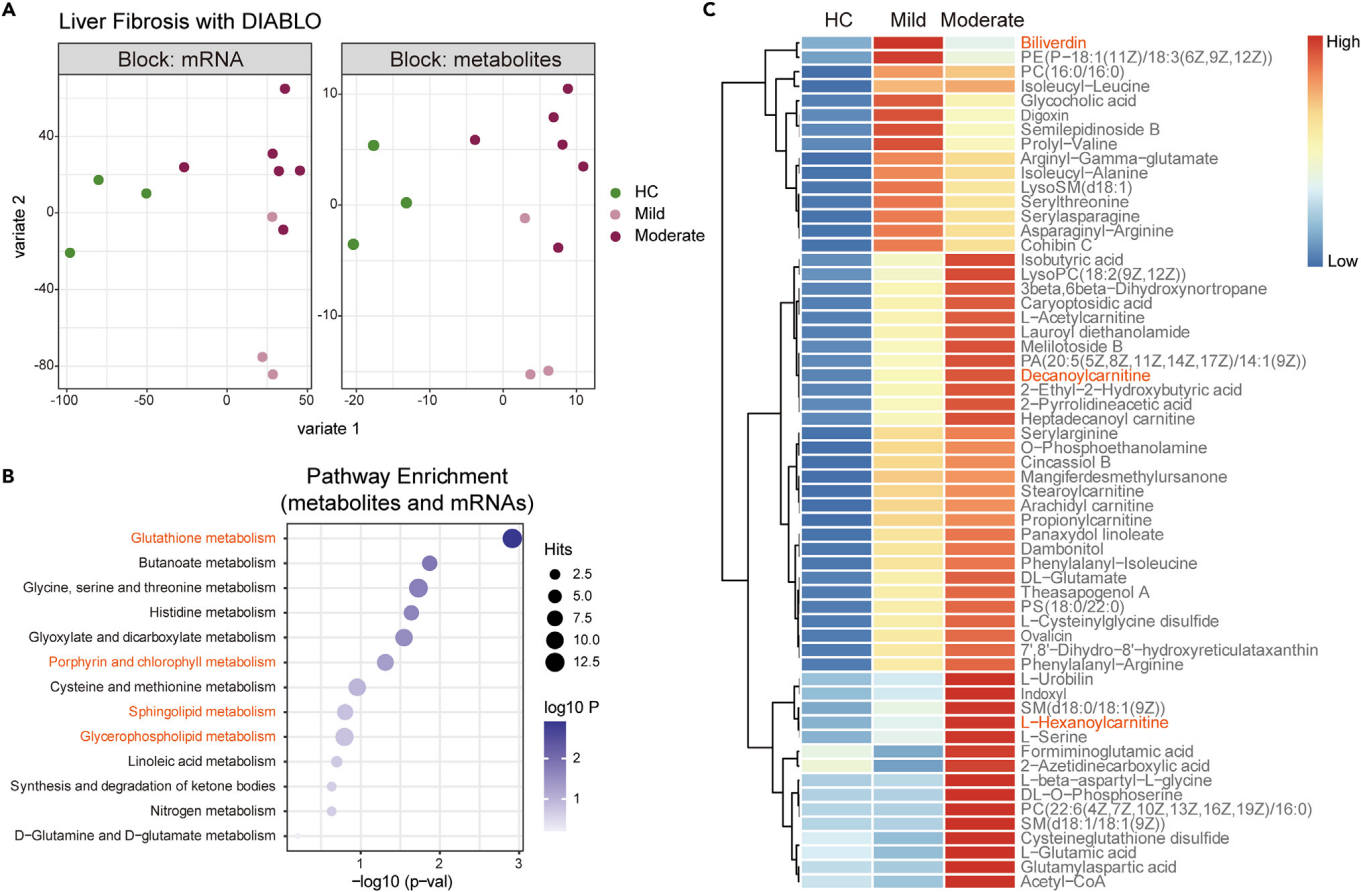
Multi-omics analysis identifies elevated metabolites in fibrotic primates (A) Integration of transcriptomics and metabolomics datasets using DIABLO strategy. (B) Functional enrichment analyses using KEGG pathways for the multi-omics results in (A). (C) Heatmaps showing the changes of 59 metabolites in (B) mentioned pathways. Data are represented as Z-score-normalized metabolite levels.

To better integrate the transcriptional and metabolic profiles in the liver of fibrotic primates, we conducted a multi-omics strategy, namely DIABLO,^11^ to statistically identify functional associations between the datasets of transcriptomics and metabolomics (Figure 3A). The incorporated multi-omics analysis supported our findings that glutathione metabolism, lipid metabolism, including butanoate/glyoxylate/linoleic acid (short chain) and sphingolipids/glycerophospholipids (long chain), and porphyrin metabolism were top-ranked pathways in the liver of fibrotic primates (Figures 3B and S2).

Next, to figure out specific metabolites that are associated with liver fibrosis, we classified the fibrosis-associated metabolites by statistical analysis and determined 59 metabolites belonged to the highly represented candidates (Figure 3C). Intriguingly, we found that several types of carnitines, including L-acetylcarnitine, decanoylcarnitine, and L-hexanoylcarnitine, were all significantly increased in fibrotic livers, which were reported to promote lipid oxidation and be elevated in a variety of metabolic diseases.^12^ It is also noted that a couple of porphyrin metabolites (e.g., biliverdin and L-urobilin) were upregulated in fibrotic livers (Figure 3C).

### Evaluation of serum metabolites on the diagnosis of liver fibrosis in human patients

**Figure 4:**
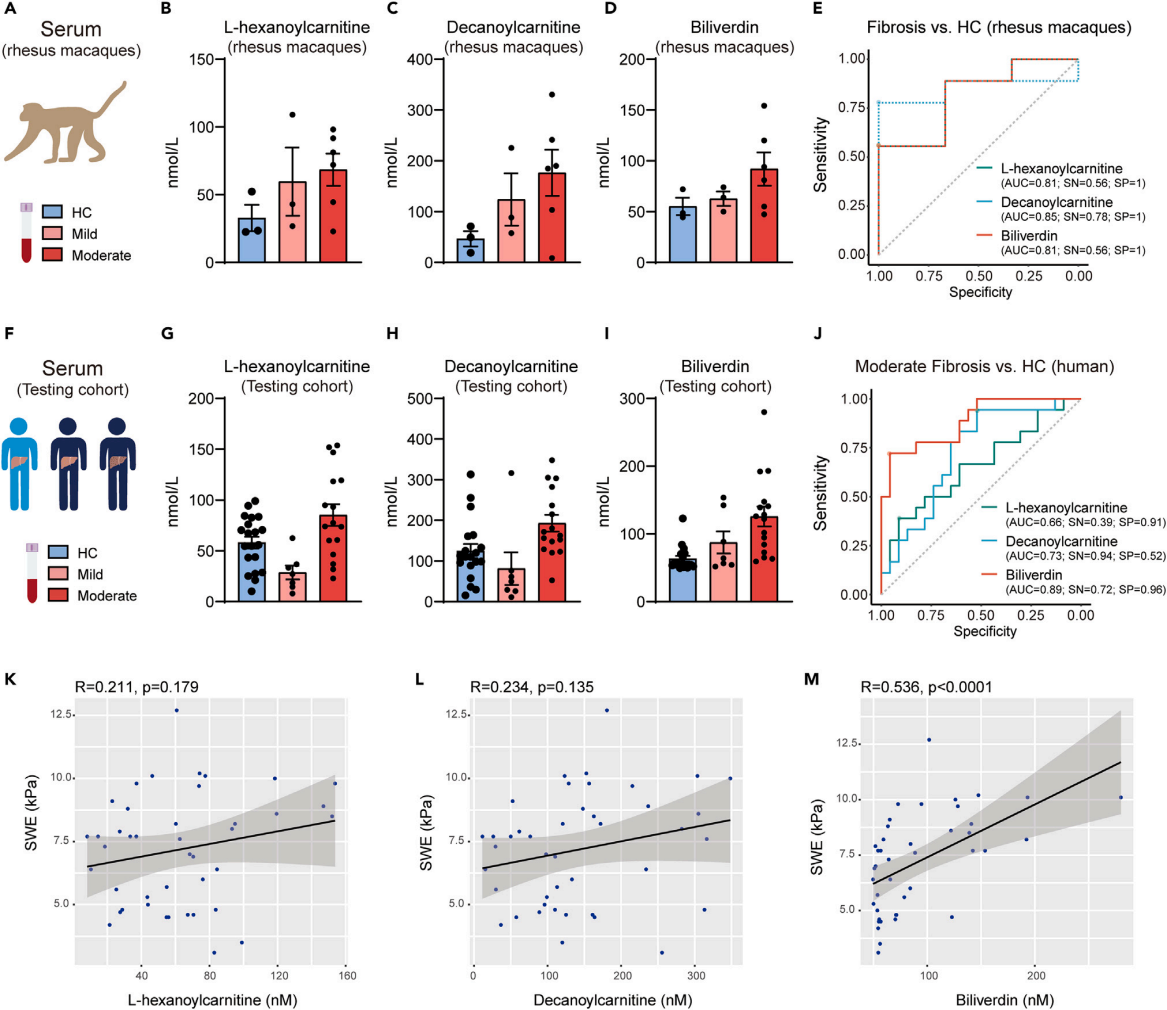
Evaluation of serum metabolites on the diagnosis of liver fibrosis in primates and human patients (A–E) Diagrams and ROC curves showing the contents of specific metabolites in the serum of fibrotic primates. (F–J) Diagrams and ROC curves showing the contents of above metabolites in the serum of patients with liver fibrosis for discrimination of liver fibrosis or moderate fibrosis from healthy controls. (K–M) Diagrams showing the correlation between metabolites in the serum of patients with the SWE values in patients.

Based on the multi-omics findings, we hypothesized that these elevated metabolites in fibrotic primates may reflect the aberrant status of liver fibrosis and thus predict disease situations (Figure 4A). As anticipated, three candidates, L-hexanoylcarnitine, decanoylcarnitine, and biliverdin were consistently increased in the serums of fibrotic primates compared to HC (Figures 4B–4D), which could predict the fibrotic status (Figure 4E). Next, we test whether these metabolites in the serum could differentiate patients with liver fibrosis from HCs (Figure 4F). Our cohort consisted of HC (n = 19), mild fibrosis (n = 7), and moderate (n = 16) fibrosis patients in chronic hepatitis B (Table 2). Results showed that all the three metabolites could distinguish moderate fibrosis patients from HC (Figures 4G–4I), with an area under the receiver operator characteristic (ROC) of 0.89 (biliverdin), 0.73 (decanoylcarnitine), and 0.66 (L-hexanoylcarnitine), respectively (Figure 4J). Moreover, these metabolites could also differentiate the whole-liver fibrosis patients (mild and moderate) from HC (Figure S3A). Of note, among all the three metabolites identified from multi-omics analysis, biliverdin exhibited the best diagnostic performance and clinical consistence with SWE (Figures 4K–4M and S3B–3D). All these data suggest that serum metabolites (e.g., biliverdin) could predict liver fibrosis in human patients.

**Table 2:** Basic information of the human cohort

	HC (n = 19)	Liver Fibrosis Patients (n = 23)		p value^a^
		Mild (n = 7)	Moderate (n = 16)	
SWE (kPa)	5.14 ± 1.07	7.66 ± 0.18	9.42 ± 1.16	<0.0001
Body weight (kg)	68.58 ± 4.99	69.14 ± 5.67	66.06 ± 4.49	0.36
Alanine transaminase (ALT, U/L)	23.79 ± 10.27	43.46 ± 13.99	75.02 ± 19.52	<0.0001
Aspartate aminotransferase (AST, U/L)	7.13 ± 19	21.06 ± 5.13	61.95 ± 16.90	<0.0001
Gamma-glutamyl transferase (GGT, U/L)	28.84 ± 11.24	24.60 ± 9.46	21.86 ± 7.96	0.12
Triglyceride (TG, mmol/L)	1.36 ± 0.48	1.94 ± 0.51	1.42 ± 0.41	0.98
Total cholesterol (TC, mmol/L)	5.00 ± 0.75	5.04 ± 0.77	4.74 ± 0.61	0.61
Total proteins (TP, g/L)	76.56 ± 6.94	74.01 ± 5.57	74.87 ± 7.02	0.85
Albumin (ALB, g/L)	43.82 ± 5.93	43.66 ± 5.03	45.48 ± 4.455	0.74
Urea (UREA, mmol/L)	5.57 ± 1.66	5.63 ± 1.54	5.70 ± 1.47	0.99
Alkaline phosphatase (ALP, U/L)	100.59 ± 24.15	81.93 ± 19.78	88.55 ± 29.23	0.44
Cholinesterase (CHE, kU/L)	9.08 ± 2.44	11.05 ± 1.49	9.87 ± 2.10	0.65
Total bile acids (TBA, μmol/L)	6.08 ± 2.96	6.71 ± 2.74	5.96 ± 2.29	1.00
Creatinine (CREA, μmol/L)	62.00 ± 9.25	66.14 ± 10.30	65.68 ± 8.57	0.57
Uric acid (UA, μmol/L)	287.39 ± 76.81	298.76 ± 69.01	258.44 ± 54.93	0.52
Total bilirubin (TBIL, μmol/L)	19.74 ± 4.69	43.17 ± 4.71	43.17 ± 7.96	<0.0001
Direct bilirubin (DBIL, μmol/L)	5.97 ± 1.22	5.91 ± 0.89	15.33 ± 2.16	<0.0001

a Indicated the p value of HC vs. Moderate.

## Discussion

The application of liquid biopsy in the diagnosis of liver diseases has been extensively studied.^13^^,^^14^ Investigation of serum metabolites may provide a potential approach to identify the underlying disease in a more convenient, safe, and repeatedly accessible manner.^6^ In this study, we performed multi-omics analysis on NHP models and revealed molecular and metabolic bases of primate fibrotic livers. Furthermore, we catapulted serum metabolites as a source of biomarkers for diagnosis of liver fibrosis in human patients.

Current knowledge to fibrotic-related reprogramming of liver metabolism is mainly derived from either rodent models or human samples, which introduces species difference or individual bias.^15^ Therefore, a better understanding of the molecular and metabolic mechanisms controlling fibrogenesis in primate models is needed to develop clinical strategies for the non-invasive assessment of liver fibrosis. To achieve the desired result of species difference in fibrotic modeling, we applied rhesus macaques as animal models by CCl_4_ treatment^10^ and demonstrated the disease relevance of differentially expressed gene transcriptions and concurrent metabolic changes. The findings that fibrotic livers displayed different metabolic patterns raised the possibility that it may distinguish liver fibrosis patients from HCs using liver-derived metabolites in the serum. The overlapping metabolic signatures between NHPs and human beings further provide the basis for the liquid biopsy by serum metabolites. In this study, we tested several differential metabolites and demonstrated that biliverdin has the best diagnostic efficiency. Biliverdin is the tetrapyrrole products of heme metabolism, which exhibits anti-oxidant and anti-inflammatory effects,^16^ and gets involved in severe acute respiratory syndrome coronavirus 2 (SARS-CoV-2) infection.^17^ The notion that biliverdin predicts liver fibrosis was consistent with the fact that increased serum biliverdin protects against liver damages.^18^^,^^19^ Here, we propose that elevated levels of biliverdin in liver fibrosis monkeys and patients (mild and moderate) may be attributed to compensatory responses that protect against liver damage in response to toxic treatments. This implies that biliverdin may be a stress-responsive metabolite, with a potential protective role in the context of liver fibrosis.

The interplay between liver fibrosis, inflammation, and metabolic changes is a multifaceted process with significant implications for the pathogenesis and progression of liver diseases. Our study established a robust liver fibrosis model using CCl_4_-induced fibrosis in rhesus macaques, incorporating assessment of inflammation to unravel the intricate connections between fibrosis, inflammation, and corresponding molecular and metabolic alterations. By incorporating serum parameters and exploring the impact of inflammation on omics analysis, we gained insights into the coordinated regulation of hepatic gene expression profiles and metabolic changes in the context of liver fibrosis. Notably, the anti-inflammatory effects of the metabolite biliverdin^20^^,^^21^ add to our understanding of the complex interplay between fibrotic changes, inflammatory responses, and metabolic alterations. These findings enhance our comprehension of liver disease mechanisms and provide a foundation for further investigations and targeted interventions.

### Limitations of the study

While our study demonstrated the diagnostic potential of serum metabolites, particularly biliverdin, in distinguishing liver fibrosis patients from HCs, we acknowledge certain practical limitations associated with the clinical detection of metabolites (e.g., biliverdin). Firstly, the availability of standardized assays for biliverdin detection is limited. Although progress has been made in this area,^22^^,^^23^ the development of widely used and validated assays for the accurate measurement of biliverdin levels in clinical samples remains a challenge. Secondly, specialized equipment and techniques may be required for the detection of biliverdin, which may not be readily available in all clinical settings.^24^ All of these necessitate further research and technological advancements to address these practical challenges and facilitate its broader utilization in clinical practice.

### Conclusions

In sum, our data outlined an interconnected network of dysregulated molecular and metabolic events in liver fibrosis models of NHPs. The extracted serum metabolites showed disease specificity and could be utilized to develop a potential liquid biopsy strategy in human patients. Our findings reveal avenues into not only the understanding of fibrosis etiology but also a non-invasive method for disease detection.

## STAR★Methods

### Key resources table

**Table:** 

REAGENT or RESOURCE	SOURCE	IDENTIFIER
**Biological samples**		

Monkey livers from CCl_4_ model	This study	N/A
Monkey serums from CCl_4_ model	This study	N/A
Human serums	This study	N/A

**Chemicals, peptides, and recombinant proteins**		

Carbon tetrachloride (CCl_4_)	Sigma-Aldrich	Cat#1601168
Olive Oil	Tocris Bioscience	Cat#O1514

**Experimental Models: Organisms/Strains**		

*Macaca mulatta*	This study	N/A

**Software and algorithms**		

R software (ver3.6.2)	R Core Team	www.r-project.org/
GraphPad Prism (version 9.1.0)	GraphPad software Inc	www.graphpad.com

### Resource availability

#### Lead contact

Further information and requests for resources and reagents should be directed to and will be fulfilled by the lead contact, Xiaolun Huang (huangxiaolun@med.uestc.edu.cn).

#### Materials availability

All unique/stable reagents generated in this study are available from the lead contact with a completed Materials Transfer Agreement.

### Experimental model and study participant details

#### Animals

This study was designed to develop non-invasive methods to detect liver fibrosis in humans. After establishment of the liver fibrosis model in rhesus macaques, the transcriptional and metabolic profiles were systematically interrogated and evaluated in rhesus macaques, and confirmed in humans. The altered metabolites in the liver were finally chosen as the indices for fibrosis detection.

In this study, twelve male rhesus monkeys (2–3 years old) were randomly divided into 3 groups that were healthy controls (n = 3), mild fibrosis (n = 3) and moderate fibrosis (n = 6). The investigators performed the animal experiments and histological staining were blinded to the group labeling.

#### Human work

The human work was performed by collecting the serum samples in liver fibrosis patients with chronic hepatitis B (n = 23, including 7 mild and 16 moderate), and healthy controls (n = 19) in Sichuan province, China. The patients were diagnosed with liver fibrosis by the Doppler color ultrasonography imaging. According to different SWE (kPa) values, these patients were split into mild and moderate sub-groups.

#### Ethical approval

The animal work was approved by the Animal Experimental Center of Sichuan Provincial People’s Hospital. The collection and handling of human serum samples were compliant with the principles of the 2013 Declaration of Helsinki. The patients enrolled in this study were provided written informed consent. The human study protocols were approved by the Institutional Review Board of Sichuan Provincial People’s Hospital. All authors had access to the study data and had reviewed and approved the final manuscript.

### Method details

#### Pharmacological modeling of liver fibrosis in *rhesus macaques*

In this study, we applied rhesus macaques as animal models of liver fibrosis by CCl_4_ treatment as we previously described.^10^ The pharmacological treatment by CCl_4_ is a canonical toxic method that resembles properties of human fibrosis quickly and reproducibly.^25^ The animal experimental protocols and procedures were reviewed and authorized by the Animal Experimental Center of Sichuan Provincial People’s Hospital. Briefly, nine male rhesus monkeys aged 2–3 years old were treated by CCl_4_ to induce liver fibrosis, and another three comparable monkeys were taken as healthy controls. All monkeys were raised in standard primate cages with the room temperature of 18°C–25°C and humidity of 40–70%. For fibrosis induction, rhesus monkeys were treated by gastric gavage of CCl_4_ (40% in olive oil; 1.5 mL/kg every 2 days), and finally anesthetized by intramuscular injection of Zoletil for liver biopsy under the guidance of supersonic Doppler (Aixplorer, France).

#### Histochemical analysis of liver fibrosis in *rhesus macaques*

For histochemical analysis, liver samples from either HC or fibrotic monkeys were fixed in 4% (w/v) neutrally buffered formalin, embedded in paraffin for hematoxylin and eosin (H&E), Sirius red and Masson stainings according to standard protocols and followed by microscopic imaging.

#### RNA sequencing of liver samples in *rhesus macaques*

Total RNA was extracted using miRNeasy mini kit (Qiagen) following the manufacturer’s instructions. RNA concentration and purity were assessed by Nanodrop (Thermo Fisher) for subsequent RNA-seq analysis were performed by Biotree company (Shanghai, China). The RNA-seq data were analyzed using the Gene Ontology (GO) and Kyoto Encyclopedia of Genes and Genomes (KEGG) pathway analyses.

#### LC-MS metabolomics of liver samples in *rhesus macaques*

Liquid chromatography–mass spectrometry (LC-MS) were applied to identify the differentiated metabolites in fibrotic livers of monkeys. LC-MS analyses were performed using an UHPLC system (Vanquish, Thermo Fisher Scientific) with a UPLC BEH Amide column (2.1 mm × 100 mm, 1.7 μm) coupled to Q Exactive HFX mass spectrometer (Orbitrap MS, Thermo) by Biotree company (Shanghai, China). All data obtained by metabolomics profiling were analyzed using MetaboAnalyst v.4.0, and pathway mapping was performed on the basis of annotated metabolic pathways in KEGG. A univariate statistical analysis was used to identify significant differences in the abundances of metabolites between fibrotic and HC groups.

#### Multi-omics analysis of transcriptome and metabolome

To identify the interrogated molecular and metabolic signatures of primate liver fibrosis, we used data integration strategies of DIABLO, which is a supervised, data-driven, hypothesis-free multi-omics integration approach.^11^

#### Serum metabolite measurements in *rhesus macaques* and human patients

To measure the serum metabolites for assessment of liver fibrosis in rhesus monkeys, serum samples were collected from peripheral venous blood for biochemical analysis. For human studies, the patients with chronic hepatitis B were enrolled in this study diagnosed as mild fibrosis (n = 7) and moderate fibrosis (n = 16) by SWE imaging, as well as comparable healthy controls (n = 19). The serum samples from participants were collected from Sichuan Provincial People’s Hospital, with informed consents prior to all study procedures. The serum samples were examined by LC-MS for targeted metabolites.

### Quantification and statistical analysis

Statistical analyses were performed using GraphPad Prism (version 9.1.0) and the R software (3.6.2). Unless stated otherwise, all bar plots show data as mean ± standard deviation (S.D). One-way analysis of variance (ANOVA) was used to determine if there were statistically significant differences between three or more groups. In all these cases, the criterion for statistical significance was p < 0.05.

## Data Availability

• All the transcriptomics and metabolomics data are available from the corresponding authors upon reasonable request. • This paper does not report original code. • Any additional information related to data analysis in this paper is available from the lead contact upon reasonable request.
