# Supplementary material for: Non-invasive assessment of liver fibrosis by serum metabolites in non-human primates and human patients
Source: iScience. 2023 Aug 3;26(9):107538. doi: 10.1016/j.isci.2023.107538 (PMC10448158; doi:10.1016/j.isci.2023.107538)
Supplement: Document S1. Figures S1–S3 [file mmc1.pdf]

iScience, Volume *26*

Supplemental information

Non-invasive assessment of liver fibrosis by serum metabolites in non-human primates and human patients

Tianhang Feng, Chunyou Lai, Qiuyun Yuan, Wanchun Yang, Yutong Yao, Mengze Du, Deyuan Zhong, Sijia Wang, Qinyan Yang, Jin Shang, Ying Shi, and Xiaolun Huang

Supplementary Figure 1

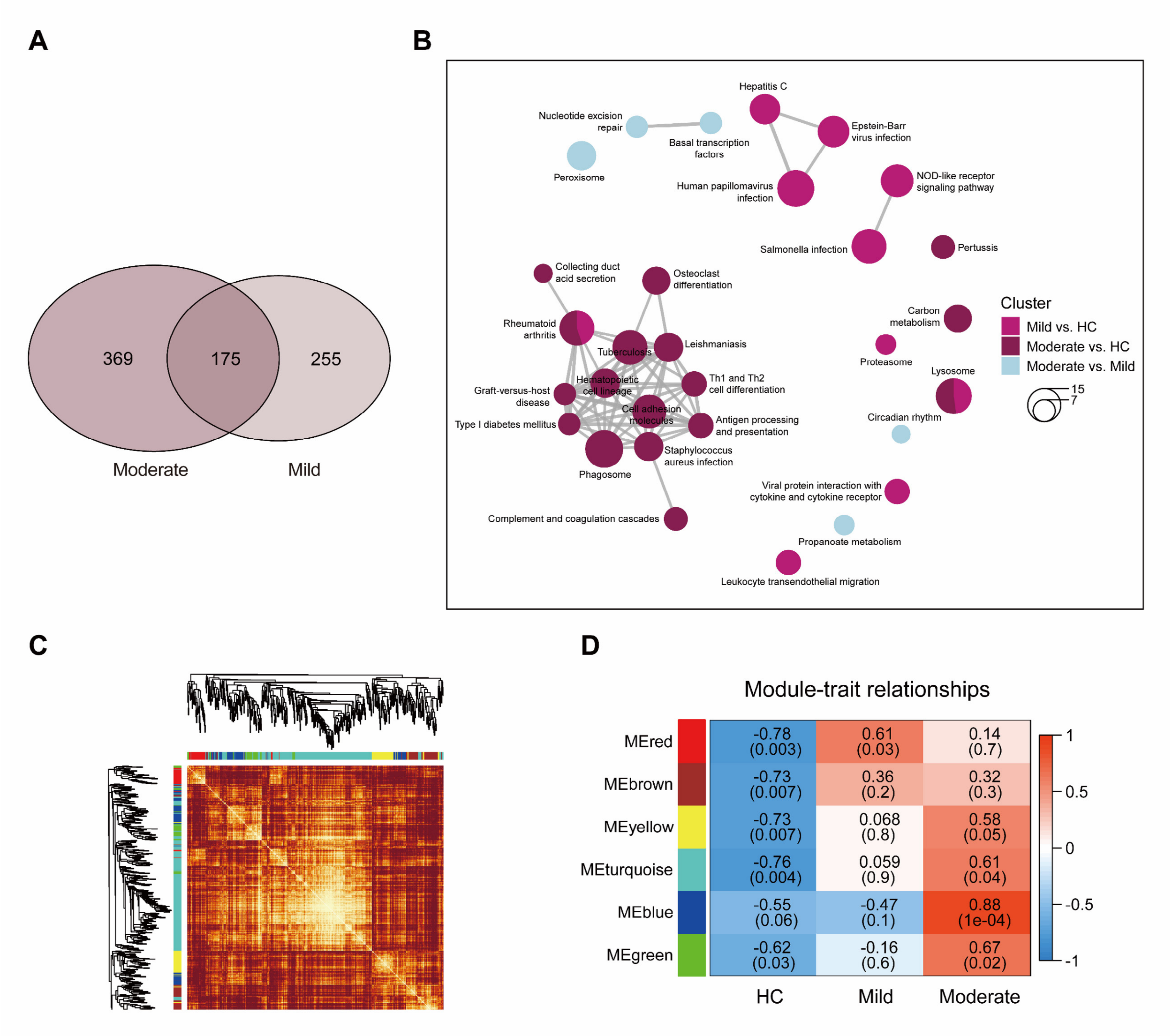

Figure S1. Transcriptomic and metabolomic signatures of liver fibrosis in non-human primates; related to Figure 2.

(A) Venn diagram showing the number of differentially expressed genes (DEGs) between mild fibrosis vs. moderate fibrosis in primates. **(B)** Core pathway mapping of gene networks that are correlated with primate liver fibrosis. Pathways correlated with mild fibrosis vs. HC and moderate fibrosis vs. HC are colored purple and dark purple, respectively. **(C-D)** The clustering dendrogram and expression heatmap of genes identifying the WGCNA modules (**C**), and module-trait correlation for the transcriptome data (**D**).

Supplementary Figure 2

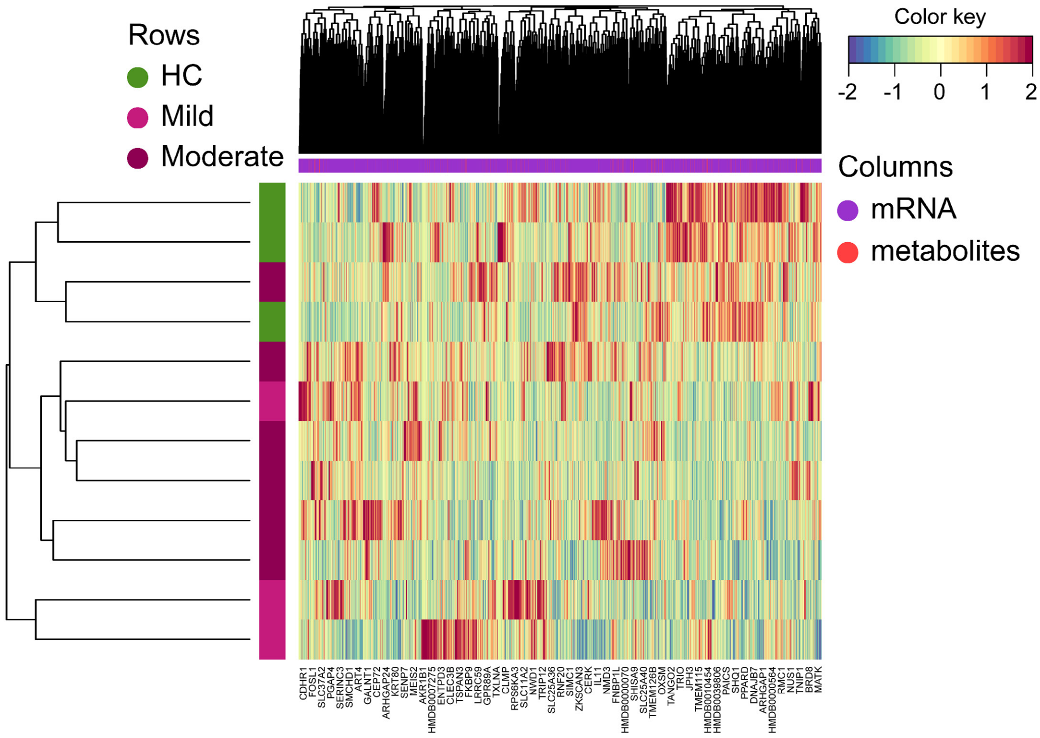

Figure S2. Multi-omics signatures of liver fibrosis in non-human primates; related to Figure 3.

Clustered image heatmap showing the multi-omics signatures of mRNA and metabolites in relation with the samples of mild and moderate fibrosis vs. HC primates.

Supplementary Figure 3

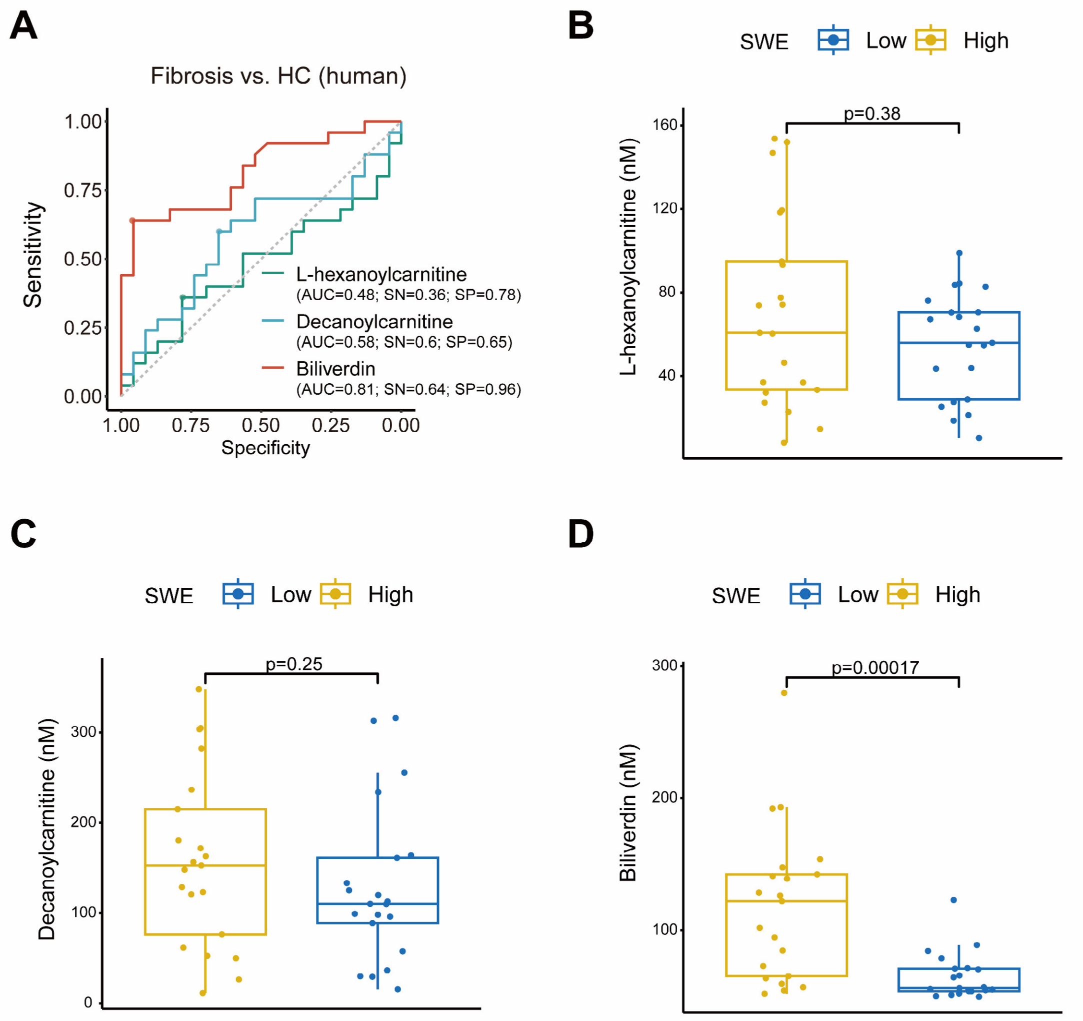

Figure S3. ROC curves in fibrotic patients; related to Figure 4.

(A) ROC curves showing the contents of serum metabolites for discrimination of liver fibrosis from healthy controls in humans. **(B)** Correlations between the SWE values with metabolite concentrations in human serums. The SWE values were cut off by the median value.
